# Hospital Administration

**Published:** 1898-10-08

**Authors:** 


### HOSPITAL ADMINISTRATION.

### ADDENBROOKE'S HOSPITAL, CAMBRIDGE.

In The Hospital of July 16, pages 276 *et seq.*, we published a report by Sir Henry Burdett on certain changes in the constitution of, and in the financial, nursing, administrative, and structural arrangements at Adden- brooke's Hospital, Cambridge, which he recommended.

The July Court of Governors referred Sir Henry's report to a Special Committee, consisting of the Lord-Lieutenant and the following members: Dr. Besant, Mr. Clark, Mr. Peck, Dr. Waraker, Mr. Whitehead, Mr. S. L. Young, Dr. MacAlister, Mr. Parker, Mr. Wherry, Mr. Humphry, and Mr. Spalding.

At the Quarterly Court, held at Cambridge on Monday, October 3rd, this committee presented a report, which was received and ordered to be printed and circulated amongst the Governors who would be summoned to deal with it at an adjourned meeting to be held on Monday, November 14th, 1898.

The report is dated September 30th, 1898, and is as follows:—

REPORT OF THE SPECIAL COMMITTEE ON SIR HENRY BURDETT'S REPORT.

"The Special Committee appointed at the Midsummer quarterly court, 1898, to consider Sir Henry C. Burdett's report on certain proposed changes in the arrangements, &c., of Addenbrooke's Hospital, beg leave to report to the president and governors as follows:—

"The committee had power to co-opt not more than three additional members, and at its first meeting it requested the President, Mr. A. P. Humphry, and Mr. W. P. Spalding to become members. The president was away from home during the summer; but the other two governors kindly agreed to join the committee. Five meetings in all have been held, and careful consideration has been given to all the points raised by Sir Henry Burdett's important report.

"It will be convenient to deal with these points in order.

"(1) Constitutional Changes.

"The committee admit the force of the arguments in favour of a change in the present method of election to vacancies on the honorary medical staff. Under the existing bye-laws what is in effect a committee of selection, consisting of the select governors and the honorary medical staff, is established for the purpose of examining into the qualifications of candidates for resident medical appointments, and of recommending the candidate it regards as the most suitable for any particular vacancy as it arises. The candidate so recommended is then submitted to the court for election, and for many years past the selection made has invariably been approved by the governors. In view of this satisfactory experience, and with the object of obviating the inconveniences and other defects of the existing practice in regard to the honorary staff, the committee think it desirable that a large and fully representative Committee of Selection should be appointed annually to consider the applications and testimonials of all candidates for vacancies on the staff, and to make definite recommendations thereupon to the court. The actual election would rest as heretofore with the governors, but in making the appointment they would have the advantage of knowing the result of a careful and impartial inquiry into the comparative merits of the several candidates.

"It appears desirable that the Committee of Selection should include the president, the members of the Committee of Management hereinafter proposed, the consulting and honorary medical officers, and three groups of seven or eight governors each, chosen annually as representative of the county, the borough, and the University respectively.

"If this arrangement were adopted, it would be advisable to declare disqualified for election any person who resorted to personal canvassing for votes in support of his candidature.

"With regard to the weekly meeting of governors, described in the bye-laws as the Weekly Board, which has hitherto managed the affairs of the hospital subject to the control of the General Court, the committee are of opinion that it would be conducive to good administration if the constitution of this meeting were more formally defined. At present, in addition to the select governors who undertake to attend, any other governors who choose may be present at the meeting and take part in all the proceedings of the board, and the actual composition of the meeting is thus liable to vary from week to week. Such variation is apt to interfere with the continuity of the administration, and to diminish the responsibility with which the governors who are willing to undertake the laborious work of management ought properly to be invested. The existing arrangement, by which twelve select governors, and seven members of the Finance Committee (some of them usually being select governors), are appointed annually, is a step in this direction; by an extension of the same principle a sufficient number of select governors, together with two members of the medical staff, making with the chairman nineteen in all, might appropriately be constituted a committee of management, and invested with the powers at present exercised by the Weekly Board, the Select Governors, and the Finance Committee. The committee of management so formed should further have the power of appointing executive and financial committees from its number, to meet more frequently than the committee itself. The office of chairman of the committee of management should be made one of dignity and responsibility, and to this end it is proposed that the appointment should be made annually by the General Court. Having regard to the importance of his functions, it is suggested that the chairman should be described as vice-president of the hospital.

"(2) Economy, Finance, and Accounts.

"The committee are gratified to observe that Sir Henry Burdett is able to report in favourable terms as to the economy with which the hospital has been managed. This fact is the more satisfactory inasmuch as within the last ten years the establishment has been enlarged in various directions to meet modern requirements, and improved accommodation for patients, resident medical officers, nurses, and ward-maids has been provided. Such extensions necessarily involve greater maintenance charges, and these can be kept within due bounds only by the exercise of constant care and forethought on the part of the administration.

"The accounts of the hospital are at present kept, so far as receipts and payments are concerned, by the treasurer, with the co-operation of his colleagues at the Bank, and the committee cordially acknowledge the value of the services thus willingly and gratuitously rendered to the hospital.

The matron keeps a detailed record of the stores received and used, the expenditure for wages, petty expenses, &c., and the receipts from probationers' fees. These records are accessible to the Weekly Board, and are periodically examined and compared with the vouchers by the Finance Committee and the auditors. Some familiarity with the system employed is, however, required to enable any governor to gain a clear idea of the actual state at a given time of the expenditure of the hospital, and much labour has had to be spent in preparing from the existing records an analytical statement of the accounts in the form desired by Sir Henry Burdett for purposes of comparison. After some consideration the committee are of opinion that it would be an advantage, both as diminishing labour and as increasing the facility of inspection and control, if the books of the hospital were kept by the method known as the 'Uniform System' of accounts. They understand that this system is used by the great majority of the important hospitals throughout the country, and its adoption would therefore make it easier from time to time to ascertain the financial position of Addenbrooke's Hospital in relation to that of others.

"The existing method of drawing up the annual financial statement at Michaelmas appears to require some explanation, in so far as the 'balance due to the treasurer' is concerned. It not infrequently happens that a considerable adverse balance is shown as 'due to the treasurer' on the year's working. But Mr. Parker has explained to the committee that the whole amount of this balance is not actually advanced at the time by the bank, and therefore does not represent a true overdraft in cash. Some of the cheques for bills passed by the Court for payment at the close of the financial year are not immediately presented at the bank, though they are rightly included in the expenditure side of the balance-sheet for the year. Before these cheques are actually presented a considerable number of subscriptions due at the beginning of October are received, and the money so paid in is consequently in hand to meet a portion of the expenditure of the preceding year. The actual sum advanced by the bank during October is thus the difference between the amounts so paid out and received, and this represents the real 'balance due to the treasurer.'

"It would in the opinion of the committee be better that the expenses incurred within the year should be completely discharged when the year's account is closed, and it is accordingly proposed to give power to the committee of management, on the recommendation of the financial committee, to provide if necessary by the sale of securities the funds required to make up any deficiency in the revenue. The expenditure during each year would then be met entirely (1) by the income received, and (2) if necessary, by the proceeds of sales during the year; the accounts would be simplified; there would be no accumulating deficit; and the state of the invested capital account would become a useful index of the financial position of the hospital. Last Lady Day the court recognised the advisability of such a step by ordering the sale of a sufficient amount of Consols to pay off indebtedness and provide a small working balance. Should the like necessity again arise, the committee of management would be enabled to adjust the account before the annual balance-sheet was finally drawn up.

"Bye-law 52, to the effect of which attention is drawn in the report, has hitherto been regarded as directing the immediate investment of all legacies, however small; while all unassigned donations, however large, have been dealt with as current revenue. It is recommended that this by-law should be rescinded, and that questions concerning the investment of both legacies and donations should be left to the Committee of Management on the recommendation of the Financial Committee. In this way a certain saving would result under the heads of brokerage and commission on the sale and purchase of stocks, and opportunities for favourable investment could be more easily taken advantage of as they occurred. The committee of management would of course report to the governors on all measures taken by them under the powers thus conferred.

"The committee have not been able to follow in detail all the figures given by Sir Henry Burdett so far as they relate to the ordinary and extraordinary income and expenditure for the three years 1894—1897. The general result at which he arrives, namely, that the receipts from all sources (including legacies and special donations) have in these years exceeded the total outgoings on maintenance and permanent improvements, appears to be warranted by the fact that, although in the period from November, 1894, to November, 1897, the sum spent on new buildings, &c., was about £5,135, the decrease in the invested capital was £764, and the increase in the 'balance due to the treasurer' was £442. But it is important to keep in mind that the receipts included several munificent and quite exceptional gifts from the president, Sir George Humphry, and others. Had it not been for these the financial position of the hospital would have been much less favourable, and the governors would have had serious difficulty, not only in undertaking the improvements referred to, but even in keeping up the establishment.

"Thus, notwithstanding the general result above mentioned, the committee feel that the falling off in the ordinary income since 1894 is very unsatisfactory, and demands the careful attention of the governors and of the general public. It would be unwise to reckon, for the maintenance of the institution, on a continued series of large donations from individual benefactors, and strenuous efforts are therefore required to increase the ordinary subscriptions and to widen the basis of public support on which the hospital must rely. It is to be hoped that the authoritative assurances as to economy of management which have now been given may lead to fuller confidence and more generous aid on the part of the residents in Cambridge and the surrounding districts.

"(3) Nursing.

"The committee find that since Sir Henry Burdett began his enquiry an important change has taken place in the arrangements for probationers, which renders his suggestions no longer apposite. Formerly, two classes were received, special probationers and nurse probationers. The former class paid in three years, for board, lodging, and training, a total sum of £98; the latter class, who had somewhat more laborious duties, a total of £40. The introduction of wardmaids, the erection of the new Peckover building, and other improvements, have made it possible to do away with the second class. Accordingly, all the probationers under training will in future have the same work and privileges, and pay the reduced fee of £60 during the first two years; while in the third year, when those who are found suitable are selected for staff duty, they will receive board, lodging, and instruction without payment. The present arrangement, which reduces the expense of training by £38, seems more favourable to the probationers than that suggested in the report, and the committee understand that it gives entire satisfaction to the select class of nurses to which the training school of Addenbrooke's is now exclusively confined. The hours of duty and the conditions of work are at present far from burdensome, and compare advantageously with those prevailing elsewhere. The fact that over two hundred applications have been received for the twelve vacancies expected to arise in the current year shows convincingly the good repute in which the training school is held.

"(4) General.

"The committee are of opinion that the improvements suggested in reference to the corridors, balconies, &c., should have the prompt attention of the Committee of Management.

"The committee have carefully considered the important proposal in the report that a resident superintendent should be forthwith appointed at a salary of, say, £250 per annum, with board and lodging. Some of the duties proposed to be laid on this officer are discharged at present by the treasurer through his staff at the bank, others by the secretary, and others again by the matron, by the visiting governors, and by the weekly board. The services at present rendered by the treasurer through his clerical staff involve no charge on the hospital funds, and those rendered by the secretary and matron are undertaken as part of their regular work. The appointment of a new officer to perform these multifarious duties would therefore add appreciably to the expenditure of the hospital, and though at a future date it will probably for many reasons be desirable to appoint a resident superintendent, the committee are not prepared to recommend this step at the present time. As they have above indicated, they do not think that the introduction of the 'Uniform System' of accounts need be delayed until such an officer is appointed, as they believe that the change from the present to the 'Uniform System' can be carried out by the co-operation of the treasurer, secretary, and matron with the committee of management.

"The committee approve the suggestion that arrangements should when practicable be made for the reception into a convalescent home of patients who no longer require active hospital treatment, but whose strength is not yet completely restored. Such arrangements would not only be beneficial to the patients, but would afford a substantial relief to the hospital, which at present is in many cases obliged to undertake their charge until they are strong enough to work.

"Recommendations.

"The committee submit to the General Court the following recommendations, which are purposely expressed in somewhat general terms in order that a decision may first be taken on the questions of principle involved. It is suggested that if these recommendations are approved the committee should be instructed to draft the necessary amendments in the bye-laws, and submit these to an adjourned court for consideration. The proposed changes would thus not come into effect until the new bye-laws were adopted.

"The committee accordingly recommend :—

"I. That a Selection Committee should be appointed annually, whose duty it should be, in case of vacancies occurring in the honorary medical staff, to recommend suitable candidates for election by the General Court.

"II. That a Committee of Management, consisting of 18 governors, together with a chairman, should be appointed annually, whose duties should include those at present assigned to the weekly board, the select governors, and the Finance Committee.

"III. That the Selection Committee should consist of about fifty members, and should include the president, the vice-president, and members of the committee of management, the consulting and honorary medical staffs, and other governors representative in equal numbers of the county, borough, and university respectively.

"IV. That a bye-law should be made providing that a personal canvassing for the votes of governors should constitute a disqualification of any candidate for a vacancy on the staff.

"V. That the Committee of Management should be instructed to appoint from their number an executive committee of twelve members to meet weekly, and a financial committee of five members to meet as often as may be required.

"VI. That a governor should be annually appointed as vice-president of the hospital, whose duty it should be to act as chairman of the committee of management.

"VII. That the investment of legacies and donations should henceforth be entrusted to the committee of management acting on the recommendation of the financial committee, and that Bye-law 52 should according be rescinded.

"VIII. That the Committee of Management should have power, if necessary, and on the recommendation of the Financial Committee, to sell out securities and apply the proceeds to make up any deficiency in revenue which may occur.

"IX. That the officers concerned with the accounts of the hospital should be instructed to bring into use as soon as may be practicable the 'Uniform System' of hospital accounts.

"X. That the committee be instructed to prepare and submit to the court at its next meeting the amendments in the bye-laws necessary to give effect to the resolutions now adopted.

"Donald MacAlister, Chairman."

Dr. MacAlister, the chairman of the Special Committee, having submitted the above report, moved that it be received and circulated, and that this Court be adjourned to November 14th, 1898, for its further consideration. In an able and moderate speech he fully explained the bearings of the proposed changes and the various recommendations of the committee, and pointed out that Sir Henry's recommendations as to the nurses had been met by a reduction in the fees paid by probationers from a total sum of £98 to a total sum of £60, as explained in the report. The convalescent arrangements were in a forward condition, whereby it was hoped that all suitable cases arising amongst the patients of Addenbrooke's Hospital would be transferred to a convalescent home, as and when they were ready for such treatment. Dr. Latham, the senior physician, seconded the resolution, and expressed a hope that the committee's recommendations would be approved. He pointed out that it would be desirable that the new bye-laws should be carefully drafted, so as to avoid many difficulties which must otherwise arise. He further expressed the opinion that 50 was too large a number for the Committee of Selection recommended by the Special Committee; he seemed to base this objection on the ground that the medical staff had heretofore always acted as a committee of selection, and that whenever their recommendations were unanimous, the Court of Governors had always upheld their decisions. When it was pointed out to Dr. Latham that the functions of the medical staff, so far as they related to the examination of the qualifications of each candidate and to the report to the Committee of Selection, would in no way be interfered with by the proposed changes in the bye-laws, he expressed himself satisfied. Several of the governors spoke in support of the committee's recommendations, and Dr. MacAlister's resolution was carried unanimously. Sir Henry Burdett gave notice that he should move at the adjourned Court that the Committee of Management be empowered to elect a general superintendent of the hospital so soon as may be practicable. Mr. Parker, the acting treasurer of the hospital, gave notice that he should second Sir Henry's resolution.

